# A Short Intervention Followed by an Interactive E-Learning Module to Motivate Medical Students to Enlist as First Responders: Protocol for a Prospective Implementation Study

**DOI:** 10.2196/24664

**Published:** 2020-11-06

**Authors:** Laurent Suppan, Tara Herren, Victor Taramarcaz, Simon Regard, Sébastien Martin-Achard, Ido Zamberg, Robert Larribau, Marc Niquille, Francois Mach, Mélanie Suppan, Eduardo Schiffer

**Affiliations:** 1 Division of Emergency Medicine Department of Anesthesiology, Clinical Pharmacology, Intensive Care and Emergency Medicine University of Geneva Hospitals and Faculty of Medicine Geneva Switzerland; 2 Save a Life Swiss Emergency Responders Association Geneva Switzerland; 3 Division of Anesthesiology Department of Anesthesiology, Clinical Pharmacology, Intensive Care and Emergency Medicine University of Geneva Hospitals and Faculty of Medicine Geneva Switzerland; 4 Unit of Development and Research in Medical Education Faculty of Medicine University of Geneva Geneva Switzerland; 5 School of Education Johns Hopkins University Baltimore, MD United States; 6 Cardiology Department University of Geneva Hospitals and Faculty of Medicine Geneva Switzerland

**Keywords:** basic life support, cardiopulmonary resuscitation, medical students, undergraduate medical education, out-of-hospital cardiac arrest

## Abstract

**Background:**

In Geneva, Switzerland, basic life support (BLS) maneuvers are provided in only 40% of out-of-hospital cardiac arrests (OHCAs) cases. As OHCA outcomes are markedly improved when BLS maneuvers are swiftly applied, a “first-responder” system was introduced in 2019. When emergency dispatchers identify a possible OHCA, first responders receive an alert message on a specific app (Save-a-Life) installed on their smartphones. Those nearest to the victim and immediately available are sent the exact location of the intervention. First-year medical students only have limited knowledge regarding BLS procedures but might nevertheless need to take care of OHCA victims. Medical students responding to out-of-hospital emergencies are off-duty in half of these situations, and offering junior medical students the opportunity to enlist as first responders might therefore not only improve OHCA outcomes but also foster a greater recognition of the role medical students can hold in our society.

**Objective:**

Our aim is to determine whether providing first-year medical students with a short intervention followed by an interactive e-learning module can motivate them to enlist as first responders.

**Methods:**

After obtaining the approval of the regional ethics committee and of the vice-dean for undergraduate education of the University of Geneva Faculty of Medicine (UGFM), 2 senior medical students will present the project to their first-year colleagues at the beginning of a lecture. First-year students will then be provided with a link to an interactive e-learning module which has been designed according to the Swiss Resuscitation Council’s first aid guidelines. After answering a first questionnaire and completing the module, students will be able to register for practice sessions. Those attending and successfully completing these sessions will receive a training certificate which will enable them to enlist as first responders. The primary outcome will be the proportion of first-year medical students enlisting as first responders at the end of the study period. Secondary outcomes will be the proportion of first-year medical students electing to register on the platform, to begin the e-learning module, to complete the e-learning module, to register for practice sessions, to attend the practice sessions, and to obtain a certificate. The reasons given by medical students for refusing to participate will be analyzed. We will also assess how comfortable junior medical students would feel to be integrated into the first responders system at the end of the training program and whether it affects the registration rate.

**Results:**

The regional ethics committee (Req-2020-01143) and the UGFM vice-dean for undergraduate education have given their approval to the realization of this study, which is scheduled to begin in January 2021.

**Conclusions:**

This study should determine whether a short intervention followed by an interactive e-learning module can motivate first-year medical students to enlist as first responders.

**International Registered Report Identifier (IRRID):**

PRR1-10.2196/24664

## Introduction

In Switzerland, as in many other countries, junior medical students only possess limited knowledge regarding basic life support (BLS) procedures [1-6]. Medical students are often off-duty when exposed to out-of-hospital cardiac arrests (OHCAs) or to other emergency situations [7]. Although they feel that the public expects them to respond to such emergencies, medical students consistently report that they are not sufficiently prepared [7,8]. There is little doubt that they could benefit from adequate training in BLS maneuvers and use of automated external defibrillators (AEDs) as these procedures significantly increase survival rates and neurological outcomes after OHCAs [9]. Some systems have strived to overcome this issue, and some medical schools have promoted a mutually beneficial collaboration with ambulance services by offering medical students the opportunity to become first responders [10].

Offering junior medical students the opportunity to enlist as first responders could increase the sheer number of first responders. This could consequently lead to a significant improvement in OHCA outcomes, as BLS was provided in less than 40% of OHCA cases between 2009 and 2012 in Geneva, Switzerland [11]. As an added benefit, this approach might foster a greater recognition of the role medical students can hold in our society, thereby increasing their global motivation toward their studies and building their future medical identity.

**Figure 1:**
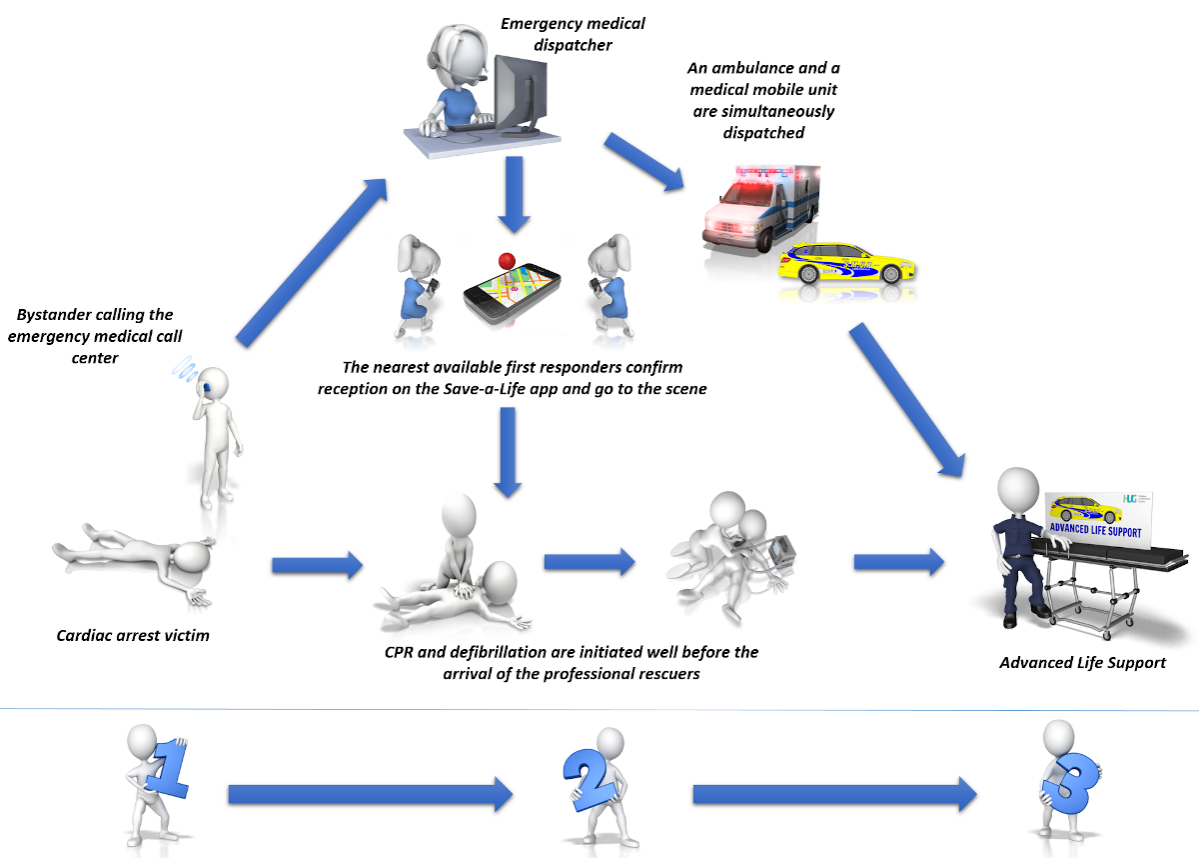
First responder system in Geneva, Switzerland. CPR: cardiopulmonary resuscitation.

Important variations in first responder systems can be observed across different jurisdictions and regions [12,13]. In Geneva, all first responders have been certified as full-fledged BLS–AED providers according to the Swiss Resuscitation Council’s (SRC) guidelines or to other certified training procedures. They can be either lay or professional rescuers (firefighters, policemen, physicians, paramedics, or members of other such professions). In this system, all medical emergencies are assessed by professional dispatchers (all with a nursing or paramedic background) working for the emergency medical call center. These dispatchers carefully assess each situation and decide whether first responders should be sent. This decision is even more important given the current COVID-19 (coronavirus disease 2019) context, as unnecessarily exposing first responders, who might be lay people, to a potentially contagious patient should be avoided as often as possible. When an alarm is sent by emergency medical call center dispatchers, all first responders receive a notification through a specific app (Save-a-Life) installed on their smartphones (Figure 1). The first responders who are both nearest to the victim and available at the time of the alert receive information regarding the exact location of the intervention. These first responders are then expected to swiftly find and quickly assess the victim, initiate the appropriate maneuvers, and retrieve the nearest available AED, the location of which is also given through the app.

Given the high workload bearing upon junior medical students, and in the context of the COVID-19 pandemic, a flexible approach based on an inverted classroom principle could prove successful. Distance learning through electronic learning (e-learning) methods has been shown to improve knowledge acquisition and user satisfaction and could efficiently promote engagement [14-16]. Such methods are particularly well adapted to the current context [17].

Our aim is to determine whether providing first-year medical students with a short intervention followed by the possibility of following an interactive e-learning module could motivate them to enlist as first responders.

## Methods

### Study Design

After obtaining the approval of the regional ethics committee (Commission Cantonale d’Ethique de la Recherche) and of the vice-dean for undergraduate education of the University of Geneva Faculty of Medicine (UGFM), senior medical students from the Geneva Medical Students’ Association (Association des Etudiants en Médecine de Genève [AEMG]) will present the project to first-year medical students at the beginning of a lecture. In Geneva, medical students follow a 6-year curriculum before graduating, and first attend BLS–AED courses during their second year [18]. Medical and dental medicine students share a common pathway during the first 2 years of this curriculum. The AEMG senior medical students will be fourth- or fifth-year students who are already certified as BLS–AED instructors. After their short intervention, they will provide their younger colleagues with a link to an interactive e-learning module. This module has been designed according to the SRC’s guidelines for the training of BLS–AED providers [19] and will be adapted to take the COVID-19 pandemic guidelines into account [20].

Before accessing the e-learning module, students will have to register on a dedicated website. They will then be required to complete a short questionnaire designed to gather demographic data, to assess their baseline knowledge, and their previous qualifications in the field (Table 1). The CHERRIES guidelines [21] will be used to design the questionnaire and to report its results. Students unwilling to register will still be offered the possibility to follow the e-learning module.

**Figure 2:**
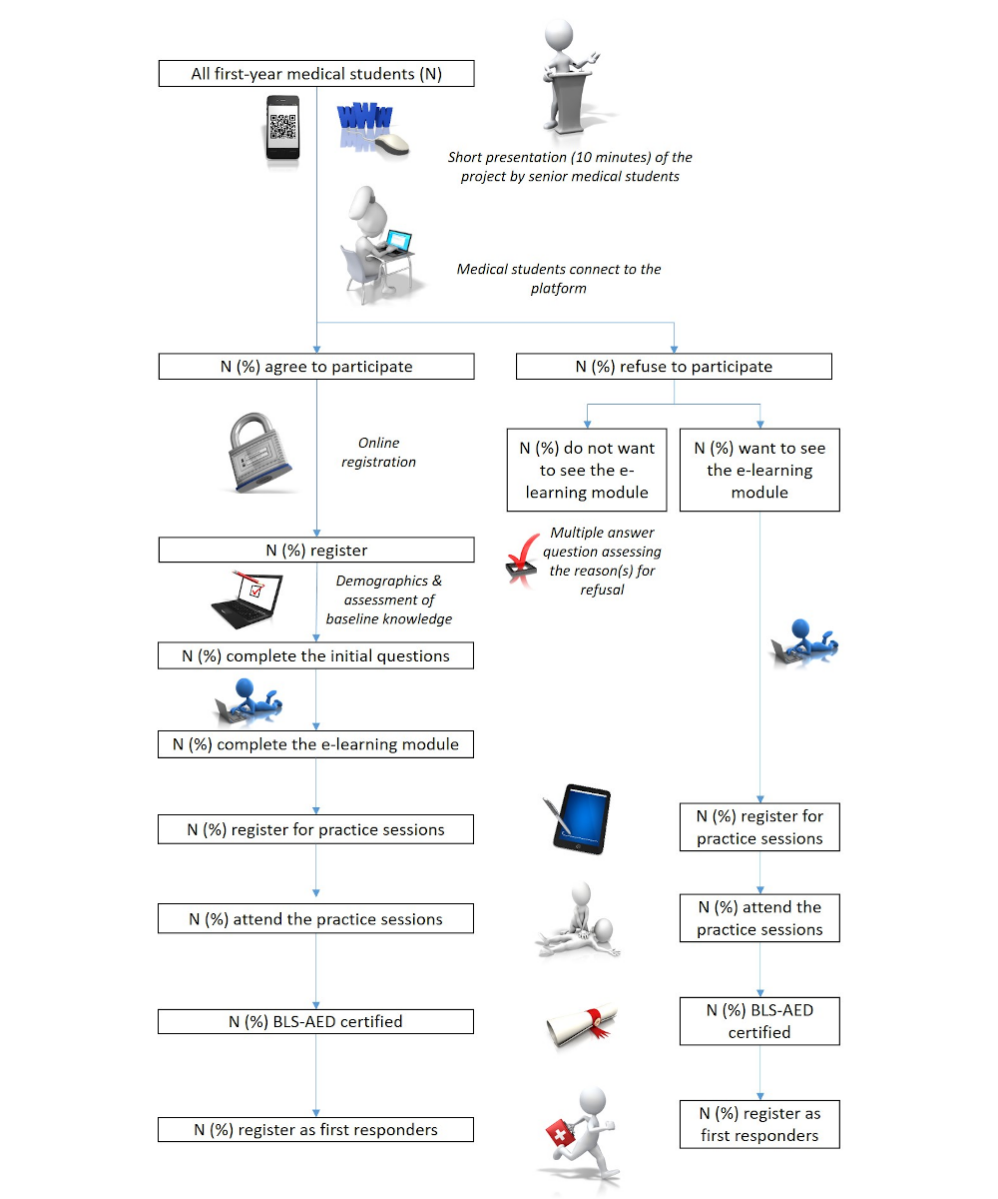
Study design.

Upon completion of the e-learning module, and whether or not they have registered on the website, first-year medical students will be offered the option to register for practice sessions. Students attending and successfully completing these sessions will receive a course certificate which will be valid for 1 year and allow them to register as first responders (Figure 2).

**Table 1:** Survey structure and questions.

Survey page, field, and question			Type of question	
**Introduction**				
	**Consent**			N/A
		Reason for refusal (if applicable)		MAQ^a^
	**Attrition**			
		N/A^b^		
**1**				
	**Demographics**			
		Year of birth		Open (Regex^c^)
		Gender		MCQ^d^
		Medical or dental medicine student		MCQ
		Previous qualifications		MCQ
		Former student or graduate of another health care profession		MCQ
		Target specialty		MCQ
**2**				
	**General BLS^e^ knowledge**			
		Ever heard of BLS or ACLS^f^ before		Yes/No
		Meaning of “AED^g^”^h,i^		Open
		Year of the last BLS guidelines update		Open (Regex)
		Phone number of the emergency medical services dispatch center^h,j^		Open
**3**				
	**Prior BLS experience**			
		Current or past: (1) student of another healthcare profession; (2) BLS instructor; (3) professional rescuer		MAQ
		Prior BLS training		Yes/No
		Wish to be trained, or more trained, in BLS procedures		Yes/No
**4**				
	**Specific BLS knowledge**			
		Criteria used to recognize out-of-hospital cardiac arrest^h^		MAQ
		BLS sequence^h^		Ordering
		Artery for pulse assessment^h^		MCQ
		Compression depth^h^		MCQ
		Compressions-to-ventilations ratio^h^		MCQ
		Compression rate^h^		MCQ
		Compression-only cardiopulmonary resuscitation^h^		Yes/No
		Treatment of a choking patient, conscious, unable to either cough or talk^h^		MCQ
		Self-assessed confidence in the ability to perform resuscitation		1-10 Likert scale

^a^MAQ: multiple answer question (more than 1 answer accepted).

^b^N/A: not applicable.

^c^Regex: regular expression validation.

^d^MCQ: multiple choice question (only 1 answer accepted).

^e^BLS: basic life support.

^f^ACLS: advanced cardiovascular life support.

^g^AED: automated external defibrillator.

^h^Questions used to assess the baseline knowledge (score out of 10 questions).

^i^All answers containing “defibrillator” in English or in French will be considered as correct (not case sensitive, spelling mistakes accepted).

^j^Acceptable answers: 112, 144, and 911 which all work in Geneva, Switzerland. 144 is the official Swiss number.

### Enrolment and Consent

A short presentation of the study lasting approximately 10 minutes will be made to first-year UGFM medical students by 2 AEMG senior medical students. First-year medical students will then be provided with a regular URL as well as with a QR code to access the online platform.

Information regarding the study’s purpose and its estimated duration will be displayed on the main page. Medical students willing to participate will then have to register on the platform using a valid email address. An electronic consent form will be displayed along with the registration form, and registration will be considered as consent to participate. Participants refusing to participate will be prompted to enter the reason for this refusal. Identity and contact details of the investigators will be given, and information regarding data handling will be provided in accordance with the European General Data Protection Regulation (GDPR) [22]. No financial incentive will be given to promote participation.

### Sample Size, Inclusion, and Exclusion Criteria

At UGFM, 521 first-year medical students are currently registered and will represent a convenience sample. First-year medical students who are already registered as first responders will be excluded.

### Online Platform, Initial Questionnaire, and Learning Material

An internet platform will be developed under the Joomla 3.10 content management system (Open Source Matters) [23].

After registration, the students will be shown a first questionnaire designed to gather demographic data and to assess their baseline knowledge. This questionnaire is adapted from a recent study by Sturny et al [6], which used questions prepared according to the 2015 International Consensus on Cardiopulmonary Resuscitation and Emergency Cardiovascular Care Science With Treatment Recommendations [24].

Given the context of the COVID-19 pandemic, the e-learning module will be adapted from an already existing module used to teach BLS–AED courses to UGFM second-year medical students according to a “flipped-classroom” principle.

### Practice Sessions and Certification

Upon completion of the e-learning module, first-year medical students will be able to register for practice sessions. These sessions will last 1 hour and will be delivered by senior medical students. All these senior medical students will be required to possess a BLS–AED instructor certificate delivered by an SRC-certified instructor.

Upon successful completion of the practice session, first-year medical students will receive a certificate enabling them to enlist as first responders.

### Data Collection, Outcomes, and Statistical Analysis

All data will be stored on an encrypted MySQL compatible database (MariaDB 5.5.5, MariaDB Foundation) located on a Swiss server.

The primary outcome will be the proportion of first-year medical students enlisting as first responders at the end of the study period. Secondary outcomes will be the proportion of first-year medical students electing to register on the platform, to begin the e-learning module, to complete the e-learning module, to register for practice sessions, to show up to the practice sessions, and to obtain a certificate. The reasons given by medical students for refusing to participate will be analyzed. We will also assess how comfortable junior medical students would feel to be integrated to the first responders system at the end of the training program and whether it affects the registration rate.

These proportions will be displayed using descriptive statistics (n [%]). Student *t* test will be used to determine a potential association between baseline knowledge and the probability of obtaining a certificate and of enlisting in the first-responder system. The same test will be used to determine whether students who feel more comfortable after their training are more likely to enlist. A sensitivity analysis will be performed according to whether the students immediately agreed to participate and register on the platform or if they rallied the study after seeing the e-learning module.

## Results

This study protocol has been submitted to the local ethics committee (Req-2020-01143), which issued a declaration of “no objection” as such studies do not fall within the scope of the Swiss Federal Act on Research involving Human Beings [25].

The vice-dean for undergraduate education of the UGFM has also approved the protocol and its underlying concept.

The study is scheduled to begin in January 2021. After discussion with the different stakeholders, we estimate that the procedure would be successful if 10% of first-year medical students had enlisted as first responders at the end of the study period.

## Discussion

### Main Considerations

The success of the approach described in this study protocol will depend on numerous factors. The short intervention performed by senior medical students will be of particular importance, as it will condition whether first-year medical students choose to connect to the online platform. As participation will not be compulsory, junior medical students might more easily heed the advice of their more senior colleagues rather than the recommendation of the teaching staff or of senior physicians [26-28].

The interactivity of the e-learning module will also be an important factor. E-learning is a generic term covering many different kinds of electronic teaching and learning materials [29]. Some e-learning methods have proven more successful than others, and enhanced interactivity has been postulated to increase learner engagement and knowledge acquisition [14-16]. The existing version of the e-learning module will therefore be adapted not only to comply to the COVID-19 guidelines [20], but also to improve its interactivity. Although this will most probably contribute to improving knowledge acquisition, practice sessions will nevertheless still be needed as practical skills can hardly be acquired through e-learning alone [30,31].

The practice sessions will need to be carefully planned. Once again, instructors should be senior medical students rather than senior physicians, as this will be less intimidating to junior medical students [26]. Combining e-learning with hands-on practice will result in a blended learning method which should enhance knowledge and skill acquisition [32,33]. Blended learning has also been shown to significantly increase motivation in undergraduate students [34]. This learning method will also allow students to spend less time in direct contact with an instructor, and courses including 45-minute practical sessions have been shown to be at least as effective as classical 6-hour courses [35]. This rather short duration should help further promote participation and limit attrition. Despite these advantages, specific safety measures, including frequent handwash and keeping a safe distance between participants, will have to be enforced given the COVID-19 pandemic context [20].

Some limitations can already be pointed out. First, the short intervention which will be used to motivate junior medical students to connect onto the web platform might not be followed by all first-year students. The participation rate might therefore be underestimated as some potential participants will not get the information and the link to the platform. Moreover, though the medical students who will follow the e-learning module without registering on the platform will still be able to register for practice sessions, we will not be able to gather data regarding their baseline knowledge. This might lead to a bias as we will aim to identify a potential association between baseline knowledge and the probability of obtaining a certificate and of enlisting in the first-responder system. A sensitivity analysis will therefore be performed to assess for such a bias. Finally, though we will strive to assess the reasons underlying the refusals to follow this learning path, we will only be able to analyze the data entered by the students who have elected to browse the study website. We might therefore miss some important clues as to how the attention of these students could be drawn.

### Conclusion

Offering junior medical students the opportunity to enlist as first responders might not only improve outcomes in OHCA victims, but also foster a greater recognition of the role medical students can hold in our society, thereby increasing their motivation. This study will determine whether providing first-year medical students with an interactive e-learning module can motivate them to enlist as first responders.

## Abbreviations

AED: automated external defibrillator
AEMG: Association des Etudiants en Médecine de Genève
BLS: basic life support
CPR: cardiopulmonary resuscitation
OHCA: out-of-hospital cardiac arrest
UGFM: University of Geneva Faculty of Medicine
